# miR-872 Protection Against Renal Ischemia-Reperfusion Injury via Targeting HMOX1

**DOI:** 10.7759/cureus.89688

**Published:** 2025-08-09

**Authors:** Jiang Wei, Zhang L Feng, Cheny Y Mao, Huang Xi, Fu Ming, Chen Jun

**Affiliations:** 1 Urology, Ezhou Central Hospital, Ezhou, CHN

**Keywords:** apoptosis, biomarker, hmox1, ischemia reperfusion injury, mir-872, mirna-mrna network

## Abstract

Background

Renal ischemia-reperfusion (IR) is an inevitable process in kidney transplantation and a main cause of acute kidney injury (AKI). The present study aimed to explore the potential miRNA-mRNA network in the development of renal IR injury (RIRI).

Methods and results

The rat IR model and cell hypoxia/reoxygenation (H/R) model were established. AKI was confirmed in Wistar rats through renal histology evaluation and blood urea nitrogen (BUN) levels. We observed a significant decrease in miR-872 in both in vivo and in vitro models. Through function assays, we confirmed the detrimental effect of miR-872 downregulation and identified HMOX1 as its direct downstream target. Using miR-872 angomir, cell apoptosis was significantly impeded.

Conclusions

The present study demonstrated that miR-872 mitigated IR injury by downregulating HMOX1, providing new avenues to treat RIRI by manipulating miRNA levels.

## Introduction

Renal ischemia-reperfusion (IR) is a severe clinical condition commonly seen in patients who have undergone major surgery, been resuscitated following cardiac arrest, or experienced microcirculation recanalization after shock, leading to acute kidney injury (AKI) [1,2]. This condition is frequently seen during major cardiovascular surgeries, cardiogenic shock, and organ transplantation [3,4]. Given the current limited treatment options for AKI, the condition can easily progress to chronic renal insufficiency [5]. The mechanisms underlying IR-induced AKI are not yet fully understood. Therefore, understanding and managing IRI are crucial to improving transplantation success rates and minimizing renal function loss.

MicroRNAs (miRNAs) are a class of short, endogenous, non-coding RNAs, typically 18-24 nucleotides in length [6,7]. Despite lacking coding potential, miRNAs can regulate gene translation by targeting the 3′-untranslated region (UTR) of target mRNA [8-10]. It has been reported that miRNAs are involved in numerous pathophysiological processes, including cell survival, stress response, cell proliferation, pyroptosis, inflammation, necrosis, apoptosis, fibrosis, and neoangiogenesis [11-13]. miR-872 is also involved in the regulation of spinal cord ischemia/reperfusion injury [14]. However, the role of miR-872 in renal IR is not yet known. As such, this study was designed to outline the role of miR-872 associated with renal IR, aiming to identify a novel diagnostic biomarker.

In this study, we confirmed that miR-872 expression was significantly downregulated in rat renal tissues. Similarly, miR-872 levels also decreased markedly in the hypoxia/reoxygenation (H/R) cell model. In function assays, we focused on its effects on IR injury (IRI). Through bioinformatic analysis, we verified that miR-872 acts as a regulator of HMOX1. The present study aims to provide a novel treatment for renal ischemia/reperfusion by modulating miRNA levels.

## Materials and methods

Animals

Male Wistar rats were purchased and housed in the Center of Experimental Animals of Wuhan University. All rats had free access to food and drink. Animal experiments were performed in accordance with the National Institutes of Health’s Guide for the Care and Use of Laboratory Animals (1996) and the Helsinki guidelines.

A total of 40 rats were randomly assigned to four groups (n = 10 each: sham group, IR group, IR + miR-872 agomir group, and IR + NC group). The miR-872 agomir was synthesized by Ribo Company (Shanghai, China). Rats were anesthetized with isoflurane (2%) administered via an endotracheal tube. Body temperature was kept at 37°C during surgery using a heating pad. A right nephrectomy was performed through a midline abdominal incision to eliminate the confounding protective effects of the contralateral functioning kidney in all rats. The IR model was induced by atraumatic clamping of the left renal pedicle for 45 minutes and 12 hours of reperfusion. miR-872 agomir (10 mg/kg) or miR-872 control was injected into the tail vein of rats for 10 days before operation.

Cell culture and transfection

Rat renal tubular epithelial cell line NRK-52E cells (ATCC, Manassas, VA) were cultured in Dulbecco's modified Eagle's medium (Thermo Fisher Scientific, Waltham, MA) with 10% fetal bovine serum (FBS) at 37°C with 5% CO2. To establish the H/R model, cells were cultured in glucose-free medium under hypoxic conditions for six hours, followed by re-oxygenation for 24 hours.

In the function assay, NRK-52E cells were transfected with miR-872 mimic, miR-872 inhibitor, or miR-NC (Ribo Company, Shanghai, China) with Lipofectamine RNAiMAX according to the kit instructions. After 24 hours of incubation, cells were transferred into standard medium and cultured for another 48 hours for further analysis.

Luciferase assay

The putative binding sites on HMOX1 3'-UTR fragment were constructed into a pmirGLO vector. HEK-293T cells were cotransfected with the Relina luciferase plasmid and miR-872 mimics or negative control. Forty-eight hours after transfection, the relative luciferase activity was evaluated using the Dual-Luciferase Reporter Assay System (Promega, Madison, WI).

Cell viability assay

Cell viability was measured using a Cell Counting Kit 8 (Dojindo, Kumamoto, Japan). In brief, cells were inoculated into a 96-well dish and incubated with 10 μL of CCK8 solution for three hours. The absorbance at 450 nm was measured using a microplate reader.

Flow cytometry

To determine the cell apoptotic rate, cells were stained with Annexin V-FITC and propidium iodide (PI) for 15 minutes. The cell apoptotic rate was analyzed using fluorescence-activated cell sorting.

Serum creatinine and blood urea assay

A blood sample was collected and centrifuged at 3000 g for 10 minutes. Serum creatinine and urea were detected by using serum creatinine and urea test kits (Beyotime, Shanghai, China), and the absorbance was measured using a spectrophotometer (T600, Persee, Beijing, China).

Malondialdehyde (MDA) and superoxide dismutase (SOD) assays

MDA and SOD levels were determined spectrophotometrically using a determination kit (Jiancheng Inc., Nanjing, China). Results were quantified using a microplate reader (V111533, Thermo Fisher Scientific, Waltham, MA) at an absorbance of 532 nm.

Histological evaluation

Kidney tissues were fixed in 4% paraformaldehyde and embedded in paraffin and sectioned into 4 μm. The sections were then deparaffinized, hydrated, and stained with hematoxylin and eosin. Histological features were photographed using a microscope (Olympus, Tokyo, Japan).

Terminal deoxynucleotidyl transferase dUTP nick end labeling (TUNEL) assay

Briefly, tissues were incubated with 50 mL of reaction buffer after fixation and washing. Following incubation, samples were stained with 3,3′-diaminobenzidine (DAB) reagent (Sigma-Aldrich, St. Louis, MO) and then mounted on gelatin-coated glass slides (Sangbio, Shanghai, China). Positive cells were photographed by a microscope (Olympus, Tokyo, Japan). Ten random fields from each section were analyzed.

Immunohistochemistry staining

Tissue sections were incubated with primary antibodies overnight after the same pre-treatment in HE staining. Primary antibodies (anti-rat Bax, ab32503, 1:250; anti-rat caspase-3, ab184787, 1:1000) were purchased from Abcam (Waltham, MA). After incubation with secondary antibodies (sc-2768; 1:500, Santa Cruz Biotechnology Inc., Santa Cruz, CA) for one hour, tissue sections were developed with DAB. Staining intensity was evaluated using a microscope (Olympus, Tokyo, Japan).

Real-time quantitative PCR

Total RNA was extracted from tissue using TRIzol reagent (Thermo Fisher Scientific, Waltham, MA). cDNA synthesis was prepared using the EasyScript One-step gDNA Removal and cDNA Synthesis Supermix kit (TransGen Biotech, Beijing, China). Analysis was performed using the TB Green PreMix Ex Taq TM kit (Takara, Kusatsu, Japan) and the Applied Biosystems 7900 (Thermo Fisher Scientific, Waltham, MA). The results were calculated using the 2-ΔΔCT method. The mRNA and miRNA expression levels were standardized to GAPDH or U6. The sequences of primers are shown in Table 1.

**Table 1:** Primer sequences for PCR

Gene	Sequence
miR-872	F: 5'-CGCCGAAGGTTACTTGTTAGTTCAGG-3'
HMOX1	F: 5’-AGTCAGGCAGAGGGTGATAGAA-3’ R: 5’-GGTCCTTGGTGTCATGGGTC-3’
U6	F: 5'-CTCGCTTCGGCAGCACA-3' R: 5'-AACGCTTCACGAATTTGCGT-3'
GAPDH	F: 5'-GTCGGTGTGAACGG GTCGGTGTGAACGGATTTG-3' R: 5'-TCCCATTCTCAGCCTTGAC-3'

Western blot analysis

Radio immunoprecipitation lysis buffer (P0013B, Beyotime, Shanghai, China) was used to harvest proteins from tissues and cells. Protein levels were quantified using a BCA kit (K763-KIT, Amresco, Solon, OH). An equal amount of protein (40 μg/lane) was separated via 10% SDS-PAGE gel (Thermo Fisher Scientific, Waltham, MA) and transferred to a polyvinylidene fluoride (PVDF) membrane. Blots were then blocked with 5% non-fat milk for one hour at 4°C, followed by incubation overnight at 4°C with primary antibodies: HMOX1 (ab189491; 1:1000; Abcam), Bax (ab32503; 1:1000, Abcam), caspase-3 (ab184787; 1:1000, Abcam), and GAPDH (sc-47724; 1:1; Santa Cruz Biotechnology Inc., Santa Cruz, CA ). Then, membranes were incubated with an HRP-conjugated secondary antibody (1:500; sc-2768; Santa Cruz Biotechnology Inc., Santa Cruz, CA). The ECL detection system (Pierce, Thermo Fisher Scientific, Waltham, MA) was used for visualization. Optical density was measured using ImageJ software version 1.48u (NIH, Bethesda, MD).

Statistical analysis

All experiments were conducted in triplicate, yielding consistent results. Statistical analysis was performed using SPSS 22.0 software. The result was presented as mean ± standard deviation (SD). Statistical significance was assessed using analysis of variance (ANOVA), followed by the Student-Newman-Keuls post-hoc test or Student's t-test for comparisons between two means. A p-value of less than 0.05 was considered statistically significant.

## Results

miR-872 downregulated in ischemic tissues and hypoxic cells

**Figure 1:**
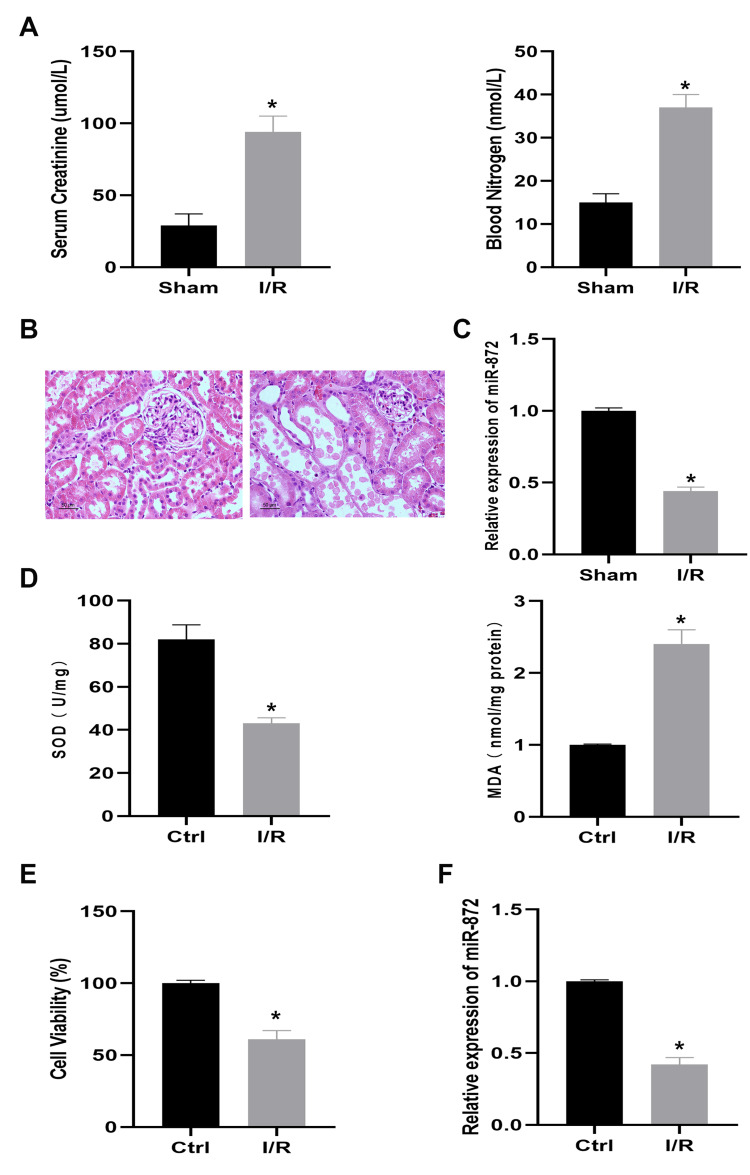
miR-872 is downregulated in ischemic tissues and hypoxic cells. (A) Serum creatinine and blood urea nitrogen levels in rat were detected. *p < 0.05 vs. sham group. (B) Hematoxylin and eosin (H&E) staining was performed to examine histological changes. (C) miR-872 level in renal tissues was analyzed by quantitative real-time polymerase chain reaction (qRT-PCR). *p < 0.05 vs. sham group. (D) Malondialdehyde (MDA) level and superoxide dismutase (SOD) activity measurements. *p < 0.05 vs. control group. (E) Cell viability level in hypoxia/reoxygenation (H/R) and control groups. *p < 0.05 vs. control group. (F) miR-872 level in H/R and control groups. *p < 0.05 vs. control group. (All experiments were conducted in triplicate.)

We first built the in vivo IR model based on rats, with animals subjected to either sham surgery or IR operation. Serum creatinine and blood urea nitrogen levels were found to be significantly elevated (Figure 1A). Pathological and morphological changes were also verified in the IR group compared to the sham group (Figure 1B). The expression level of miR-872 was then measured using quantitative real-time polymerase chain reaction (qRT-PCR), revealing a marked decrease in miR-872 levels in renal tissues of the IR group compared to the sham group (Figure 1C). To assess oxidative stress in renal tubular epithelial cells, we measured MDA levels and SOD activity in the NRK-52E cell line. The results showed a significant increase in MDA content and a significant decrease in SOD activity in the H/R group compared to the control group (Figure 1D). Additionally, cell proliferation was markedly inhibited after H/R treatment in NRK-52E cells (Figure 1E). qRT-PCR further revealed that miR-872 level was significantly downregulated in NRK-52E cells after H/R treatment (Figure 1F). These findings collectively suggest that miR-872 may play a role in renal IR injury and H/R-treated NRK-52E cells.

miR-872 promoting cell proliferation in vitro

**Figure 2:**
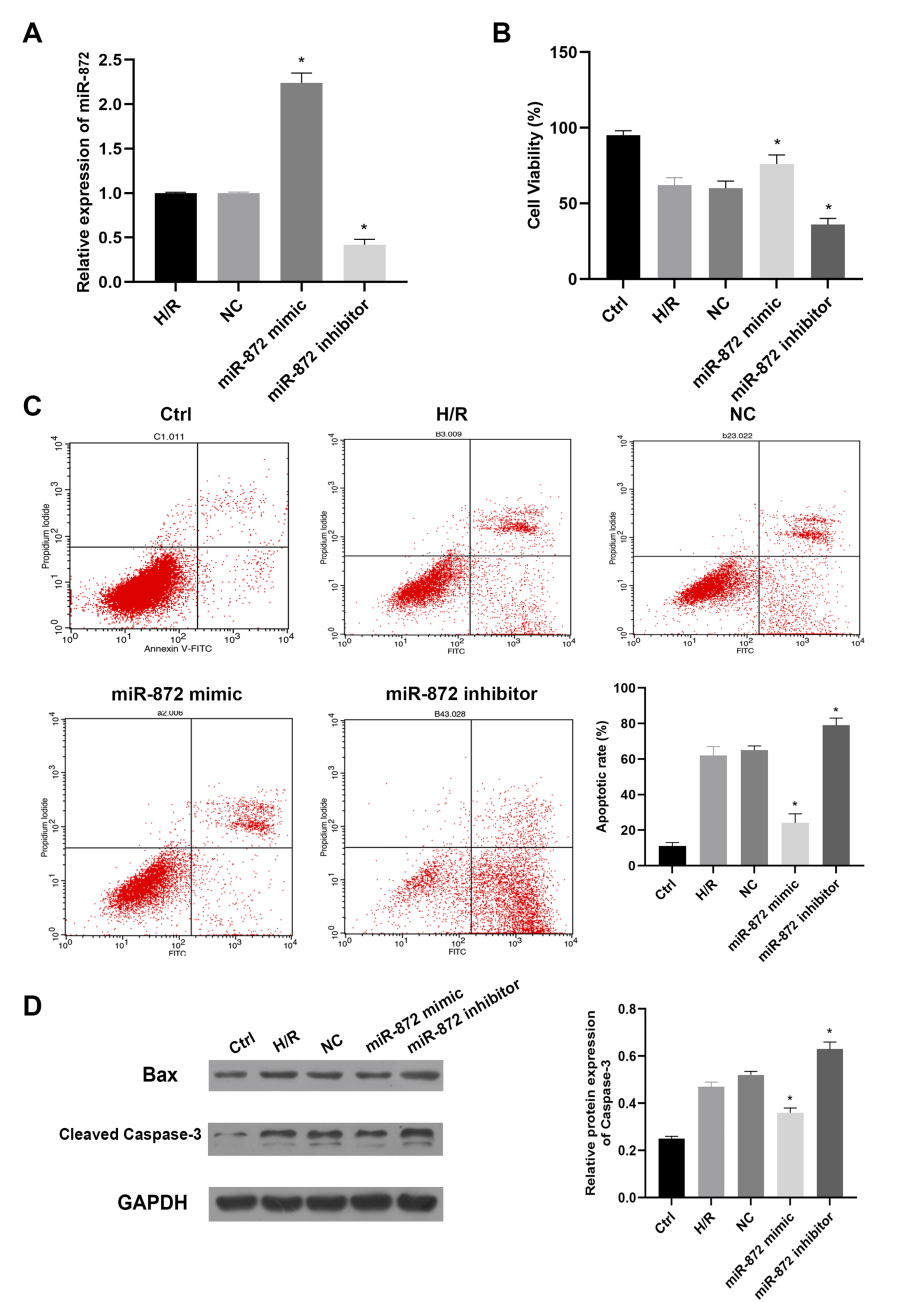
miR-872 promotes cell proliferation in vitro. (A) Transfection efficiency was verified by quantitative real-time polymerase chain reaction (qRT-PCR). *p < 0.05 vs. negative control (NC) group. (B) Effect of miR-872 on cell viability. *p < 0.05 vs. NC group. (C) Effect of miR-872 on cell apoptosis. *p < 0.05 vs. NC group. (D) Bax and cleaved caspase-3 protein expression measurements by immunoblotting analysis. *p < 0.05 vs. NC group. (All experiments were conducted in triplicate.)

To investigate the impact of miR-872 on the H/R model, miR-872 mimics or inhibitors were used to modulate miR-872 levels. Data from qRT-PCR indicated that miR-872 expression significantly increased with miR-872 mimics and decreased with miR-872 inhibitors (Figure 2A). CCK8 assay results demonstrated that transfection with miR-872 mimics markedly promoted cell viability in the H/R group compared to the control group, whereas miR-872 knockdown triggered an opposite result (Figure 2B). Data from flow cytometry revealed that miR-872 overexpression markedly impeded cell apoptotic rate, while miR-872 knockdown mildly promoted cell apoptosis (Figure 2C). Additionally, immunoblotting results revealed that miR-872 overexpression reduced the protein levels of Bax and caspase-3, whereas miR-872 knockdown showed a similar opposite trend as the former assay (Figure 2D). Taken together, the above results suggest that miR-872 can promote cell proliferation and inhibit cell apoptosis against H/R injury.

miR-872 acting as a regulator of HMOX1

**Figure 3:**
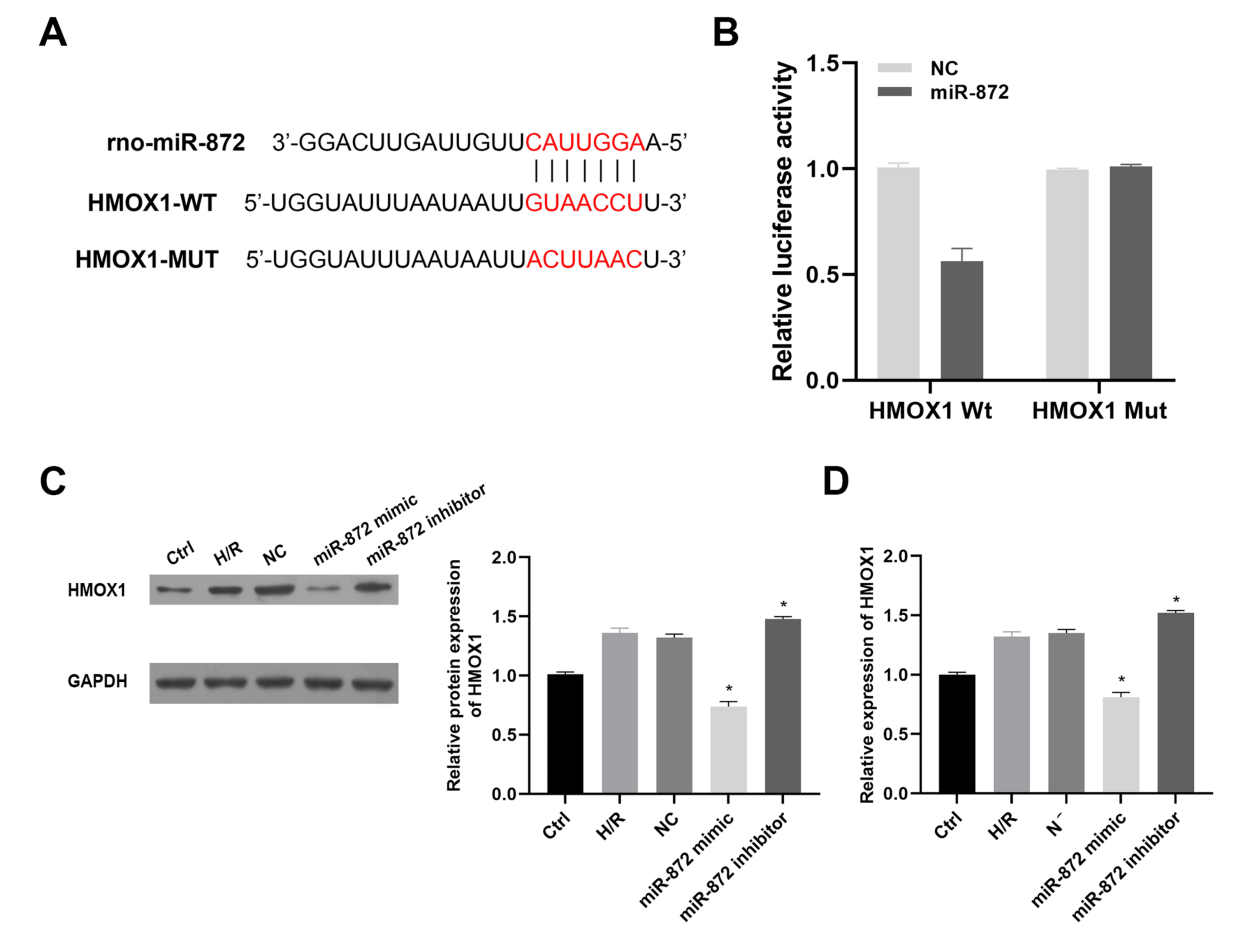
miR-872 acts as a regulator of HMOX1. (A) The putative binding sites of miR-872 on HMOX1. (B) The verification of binding correlation by dual luciferase reporter assay. *p < 0.05 vs. negative control (NC) group. (C) HMOX1 protein expression was examined by immunoblotting. *p < 0.05 vs. NC group. (D) HMXO1 mRNA level was examined by quantitative real-time polymerase chain reaction (qRT-PCR) assays. *p < 0.05 vs. NC group. (All experiments were conducted in triplicate.)

To investigate the correlation between miR-872 and HMOX1, a bioinformatic prediction was conducted via databases (TargetScan and RNAhybrid) (Figure 3A). Luciferase reporter plasmids containing either the wild-type (Wt) or mutated (Mut) 3'UTR sequence of HMOX1 were constructed. As data showed, miR-872 markedly suppressed the luciferase activity in the Wt group, while the activity remained in the Mut group. Data from immunoblotting and qRT-PCR demonstrated that miR-872 reduced both protein and mRNA levels of HMOX1, whereas miR-872 knockdown resulted in a significant increase in HMOX1 expression (Figures 3C-3D). These findings indicate that miR-872 acts as a regulator of HMOX1.

miR-872 regulating cell apoptosis in vivo

**Figure 4:**
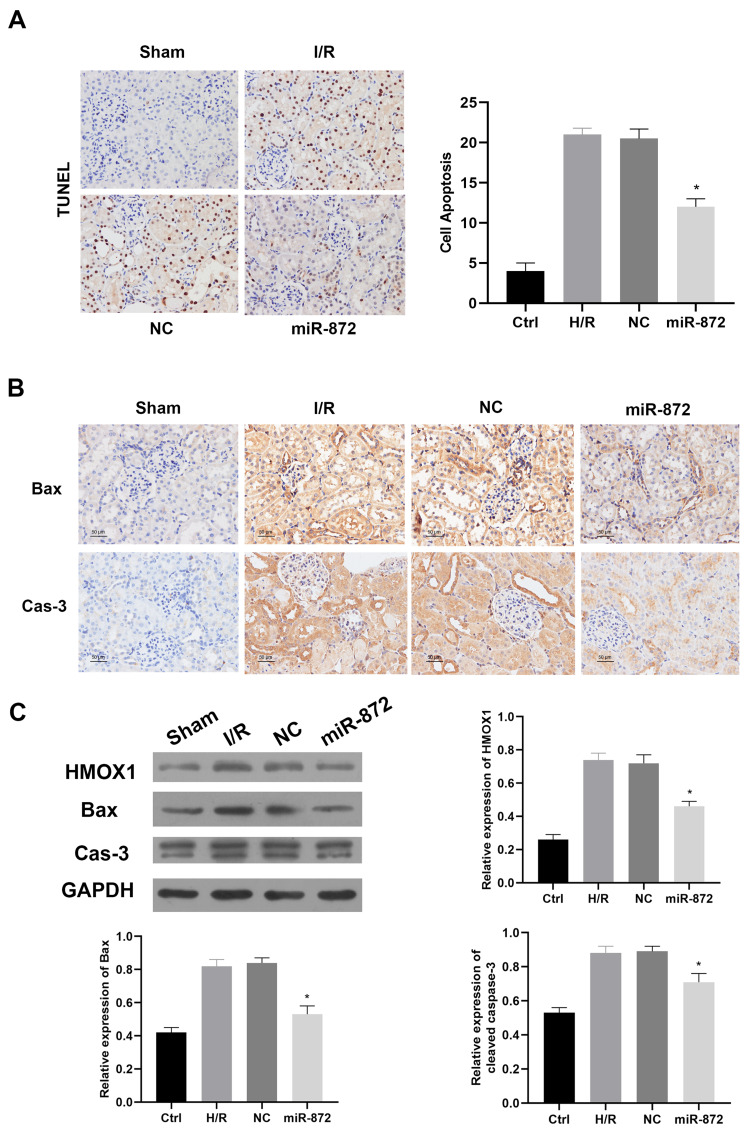
miR-872 regulates cell apoptosis in vivo. (A) Representative micrographs showing terminal deoxynucleotidyl transferase dUTP nick end labeling (TUNEL) assay of rat transfected with miR-872 angomir. *p < 0.05 vs. negative control (NC) group. (B) Representative micrographs of immunohistochemistry (IHC) staining of Bax and caspase-3 in renal tissues. *p < 0.05 vs. NC group. (C) Protein levels of HMXO1, Bax, and caspase-3 in renal tissues. *p < 0.05 vs. NC group. (All experiments were conducted in triplicate.)

To further assess the effect of miR-872 on cell apoptosis in an in vivo model, miR-872 angomir or miR-872 NC was injected intravenously via the tail vein. The TUNEL assay demonstrated that miR-872 significantly reduced IR-induced cell apoptosis (Figure 4A). Immunohistochemistry staining showed that the expression of Bax and caspase-3 obviously decreased in the miR-872 overexpressed group compared to the IR group (Figure 4B). Additionally, immunoblotting indicated a similar result that miR-872 overexpression significantly reduced the expression of HMOX1, Bax, and caspase-3 in tissues (Figure 4C). Collectively, these findings suggest that miR-872 inhibits cell apoptosis in vivo.

## Discussion

Renal IR injury is a significant cause of AKI and a major complication following renal transplantation in clinical settings [15,16]. The initial hypoxic condition, followed by reperfusion, triggers immune and inflammatory responses, leading to necrosis or apoptosis by damaging cellular DNA, proteins, and overall cellular integrity. Therefore, developing interventions to prevent ischemia-induced AKI is essential [17-19]. The present study demonstrates that miR-872 protects against oxidative stress damage in renal tissues by downregulating HMOX1. We observed that miR-872 and HMOX1 showed opposite expression levels in the in vivo IR model and in vitro H/R model. Bioinformatic analysis suggests that HMOX1 is a potential target of miR-872. In vitro functional enhancement and relative luciferase activity experiments confirmed that miR-872 directly and negatively regulates HMOX1 expression. Moreover, we found that exogenous miR-872 significantly ameliorates renal IRI in rats. These findings suggest that miR-872 is a potential biomarker for renal IRI and a novel target for AKI treatment.

Previous studies have shown that HMOX1 plays a crucial role in hypoxia and infections in myocardial osteosarcoma cells, breast epithelium during mastitis, and cardiomyocytes in sickle cell disease [20-22]. Additionally, HMOX1 has been identified as a key factor in the development of AKI [23]. In our present study, we found that HMOX1 expression levels were evidently increased in rats with renal IRI and ROS rupture. This suggests that HMOX1 plays a crucial role in oxidative stress injury associated with renal IR. Understanding the regulatory mechanisms of HMOX1 expression is therefore of great importance.

MiRNAs, small non-coding RNAs, regulate the transcription of various genes involved in apoptosis, necrosis, inflammation, and fibrosis. Over the past decade, the role of miRNAs in IRI has been extensively studied. Given the close association between HMOX1 and IR injury, targeting HMOX1 with miRNA-based therapies has garnered widespread attention. We focused on miR-872 in our study because its expression level in rat kidney tissue is negatively correlated with HMOX1 expression. By using bioinformatics analysis, HMOX1 was found to be a potential target of miR-872. This hypothesis was confirmed through miR-872 mock transfection and luciferase reporter gene experiments. These data strongly suggest that miR-872 plays a key role in oxidative stress during IRI. Our current research further shows that miR-872 overexpression induced by an agonist significantly downregulates HMOX1 in rats and ameliorates IR damage. These findings suggest that miR-872 is a promising target for developing effective treatments in renal IR injury.

## Conclusions

In this study, a bioinformatics approach identified HMOX1 as a key regulator of renal IRI through its targeting by miR-872. We explored the mechanism by which miR-872 can prevent IR damage in renal tubular epithelial cells. This is achieved, at least in part, by promoting HMOX1 downregulation.
